# Equity in Economic Evaluations of Early Childhood Development Interventions in Low-and Middle-Income Countries: Scoping Review

**DOI:** 10.1007/s10995-023-03650-3

**Published:** 2023-04-10

**Authors:** Yeji Baek, Zanfina Ademi, Jane Fisher, Thach Tran, Alice Owen

**Affiliations:** 1 School of Public Health and Preventive Medicine, Monash University, Melbourne, VIC 3004 Australia; 2 Centre for Medicine Use and Safety, Faculty of Pharmacy and Pharmaceutical Sciences, Monash University, Melbourne, Australia

**Keywords:** Equity, Cost-effective, Early childhood development, Review, Low-and middle-income countries

## Abstract

**Objectives:**

This study aimed to examine how equity is integrated into economic evaluations of early childhood development interventions in low-and middle-income countries (LMICs), and to narratively synthesize the study characteristics and findings.

**Methods:**

We conducted a scoping review by searching three electronic databases with terms including equity, early childhood development intervention, economic evaluation, and LMICs. Interventions that aimed to improve child cognitive, physical, language, motor, or social and emotional development through health, nutrition, security and safety, responsive caregiving, and early learning interventions between conception and age 8 years were considered. Studies published in English peer-reviewed journals in the year 2000 and later were included.

**Results:**

The review included 24 cost-effectiveness studies out of 1460 identified articles based on eligibility criteria. The included studies addressed health, nutrition, social protection, and water, sanitation and hygiene interventions for child development. The common type of intervention was immunization. Mostly, equity was measured using household wealth or geographic areas, and the study findings were presented through subgroup analyses. The study settings were LMICs, but most studies were conducted by research teams from high-income countries. Overall, 63% of included studies reported that early childhood development interventions improved equity with greater intervention benefits observed in disadvantaged groups.

**Conclusions:**

Consideration of equity in evaluations of early childhood interventions provides a more complete picture of cost-effectiveness, and can improve equity. Greater focus on promoting equity consideration, multi-sectoral interventions, and researchers in LMICs would support evidence-based interventions and policies to achieve equity in child development.

**Supplementary Information:**

The online version contains supplementary material available at 10.1007/s10995-023-03650-3.

## Significance

The review found that existing studies mostly measured equity by wealth groups or geographic areas, and presented their findings through subgroup analyses. The most common type of intervention was childhood immunization. The study settings were LMICs, but most studies were conducted by research teams from high-income countries. More than half of studies reported that early childhood development interventions improved equity with greater intervention benefits observed in disadvantaged groups.

The small number of relevant studies in the review highlights that more emphasis on equity integration into economic evaluation, coordinated work across multiple sectors, and strong involvement of researchers based in LMICs, are necessary to improve child development.

**Supplementary Information:**

The online version contains supplementary material available at 10.1007/s10995-023-03650-3.

## Introduction

The United Nations Convention on the Rights of the Child highlights that children have the right to good quality health care, clean water, nutritious food, a clean environment and an education, to meet their physical and mental needs, and develop their personality and talents to the full (United Nations, 1990). Early childhood development refers to children’s cognitive, physical, language, motor, and social and emotional development, between conception and age 8 years (World Health Organization et al., 2018). Scientific evidence shows that optimal early childhood development is essential to develop intellectual skills, creativity, and wellbeing across the life course, with long-term consequences for the care of the next generation and for the wellbeing of societies (Black et al., 2017; Richter et al., 2017; Walker et al., 2011). In particular, conception to age 3 years is known as the time when adverse exposures exert the greatest harm, and effective interventions return the greatest benefit (Black et al., 2017; Richter et al., 2017). However, according to the data from 94 low-and middle-income countries (LMICs) between 2010 and 2018, 37% of children under 5 years of age were exposed to risk of poor development due to malnutrition or extreme poverty, and 39% of children (36–59 months) ever attended early care and education programs (Lu et al., 2020). Substantial gaps in early childhood development indicators across country income groups, residential areas and household wealth categories were reported. Outcomes for children in urban areas or in the richest household wealth quintiles were better than those in rural areas or the lowest wealth quintile, which demonstrates disparities in child development (Lu et al., 2020).

Health equity can be defined as the absence of systematic disparities in health between more and less advantaged social groups, and equity can also mean social justice in health with a moral dimension as a broad term (Braveman, 2014; Braveman & Gruskin, 2003; Whitehead, 1992). Health equity therefore indicates the highest possible standard of health for all people with more attention paid to the needs of disadvantaged groups (Braveman, 2014). Cookson and colleagues described two main ways of using cost-effectiveness analysis to address health equity, equity impact analysis, and equity trade-off analysis (Cookson et al., 2017). However, unlike advances in diverse and complex methods to assess cost-effectiveness in health economics, relatively less effort has been made to fully incorporate equity considerations into economic evaluations. A small number of reviews have been conducted in the last decade to examine the state of integration of equity in health economic evaluations (Avanceña & Prosser, 2021; Boujaoude et al., 2018; Dukhanin et al., 2018; Johri & Norheim, 2012; Lal et al., 2018; Yang et al., 2021). The reviews concluded that feasible methods to consider equity in economic evaluations exist, yet they have not been widely used, and some challenges for application were still found including equity measurement and valuation. In addition to assessing cost-effectiveness of early childhood development interventions, equity integration will provide a clearer understanding of the broader implications of interventions. Despite the potential benefits, how equity is considered in economic evaluations of early childhood development interventions, and how specific interventions affect equity are not well understood. Therefore, this scoping review aimed to examine what methods are used for equity consideration in economic evaluations of early childhood development interventions in LMICs, and to narratively synthesize the study characteristics and findings.

## Methods

### Search Strategy with Eligibility Criteria

We used a combination of subject headings including MeSH and free text terms to cover the following concepts: (1) equity, (2) early childhood development intervention, (3) economic evaluation, and (4) LMICs. We developed the search strategies in consultation with an information analyst and searched MEDLINE (Ovid), EMBASE (Ovid) and EconLit on 13 July 2021. In addition, hand searching and citation checking were undertaken to supplement database searching. The search strategy for MEDLINE can be found in Supplementary Table 1. The study followed the Preferred Reporting Items for Systematic reviews and Meta-Analyses extension for Scoping Reviews (PRISMA-ScR) guidelines (Tricco et al., 2018). The protocol was not prospectively registered in PROSPERO as they do not accept scoping review protocols.

The key concepts with eligibility criteria are described in Table 1. The WHO has defined health equity as the absence of unfair, avoidable, and remediable differences in health status among groups of people (World Health Organization, 2021). Braveman and colleagues have stated that equity means social justice or fairness, and health equity is the absence of systematic disparities in health between more and less advantaged social groups (Braveman & Gruskin, 2003). Without limiting equity to a certain concept, since equity is a broad term, we aimed to examine how existing studies conceptualized and incorporated equity into their economic evaluations. We included studies that addressed any equity aspects such as the distribution of health outcomes by income or geographical regions. Multi-country studies were not included if they only provided country-level data without equity consideration within country. We identified early childhood development interventions based on the 2016 Lancet Early Childhood Development Series (Black et al., 2017; Britto et al., 2017). We included interventions that aimed to improve domains of child development including language, cognition, motor, social and emotional development, and psychosocial wellbeing. Accordingly, we included health, nutrition, security and safety, responsive caregiving, and early learning interventions which targeted children from conception to the age of 8 years. For study type, economic evaluations such as cost-effectiveness analysis that compared both the costs and the outcomes of at least one intervention and an alternative were included. In addition, extended cost-effectiveness analysis which examined financial risk protection benefits along with health outcomes of interventions (Verguet et al., 2016), and distributional cost-effectiveness analysis which provided the information about equity impacts and the trade-offs regarding who gained the benefits and who bore the burdens (Cookson et al., 2021) were included. We excluded review papers, commentaries and conference proceedings. LMICs were identified based on the World Bank classification as per the year of publication. For 2021 fiscal year, low-income economies were defined as those with Gross National Income (GNI) per capita US$1035 or less, lower middle-income economies were those with GNI per capita between US$1036 and US$4045, and upper middle-income economies were those with GNI per capita between US$4046 and US$12,535 (World Bank, 2020). Lastly, we included original scientific literature in English peer-reviewed journals published in the year 2000 and later. The restriction on publication period was determined because research, programs, and policies on early childhood development have advanced mostly since 2000 (Black et al., 2017) and recent systematic reviews on equity in economic evaluations only identified publications after 2010 (Avanceña & Prosser, 2021; Lal et al., 2018).

**Table 1:** Key concepts with eligibility criteria

Concepts	Descriptions	Eligibility criteria
Equity	Absence of unfair, avoidable, and remediable differences in health status among groups of people (World Health Organization, 2021)	Included studies that addressed any equity aspects
Early childhood development interventions	Interventions to improve child development outcomes such as language, cognition, motor, social and emotional development, and psychosocial wellbeing. Health, nutrition, security and safety, responsive caregiving or early learning interventions	Included studies with early childhood development interventions Interventions which targeted children under 8 years of age and their caregivers including women who were mothers
Economic evaluation	Full economic evaluations comparing both the costs and the outcomes of at least one intervention and an alternative, such as cost-effectiveness analysis	Included full economic evaluations
Low and middle-income countries	Based on the World Bank classification as per the year of publication. For instance, World Bank classification for 2021 fiscal year Low-income economics: GNI per capita US$1035 or less Lower middle-income economies: GNI per capita between US$1036 and US$4045 Upper middle-income economies: GNI per capita between US$4046 and US$12,535	Included studies in low and middle-income countries setting
Others	–	Included original scientific literature published in English peer-reviewed journals Research articles published in the year 2000 and later Excluded review papers, commentaries and conference proceedings

*GNI* Gross national income

### Study Selection, Data Extraction, and Synthesis

One reviewer (YB) screened titles and abstracts, and assessed full text based on the eligibility criteria. Other reviewers (ZA, JF, TT, AO) addressed any uncertainties.

We used a standardized form to extract study characteristics, equity measures, and results of included studies. The data extraction form was finalized after pilot testing. The following data were extracted: author, year, country, description of intervention and comparator, study design, economic evaluation type, study perspective, equity measures and methods of analysis, and results. We present findings through a narrative synthesis due to the substantial heterogeneity in study designs, settings, interventions, characteristics of participants, and outcome measures. We used Excel, Covidence and Endnote software for data management. Since scoping reviews do not aim to produce a critically appraised and synthesized answer to a particular question, and rather aim to provide an overview or map of the evidence (Munn et al., 2018), we did not assess quality of included studies’ methods or reporting practices.

### Ethical Approval

Ethical approval was not required for this review as it is based on published studies and does not draw on data contributed by patients or members of the community.

## Results

### Characteristics of Included Studies

**Fig. 1:**
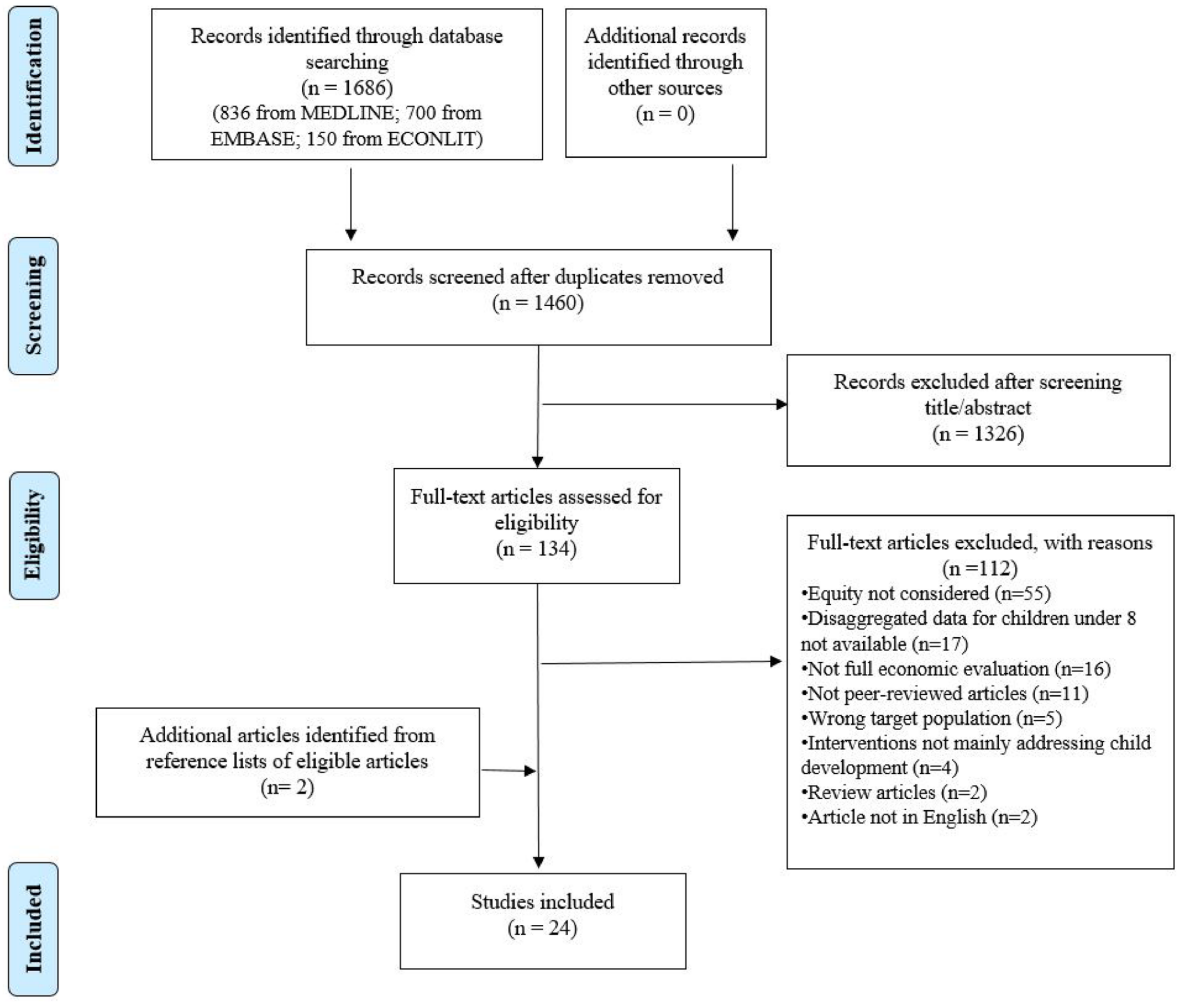
PRISMA flow diagram showing study selection

The search identified 1460 articles after removing duplicates. After screening titles and abstracts based on eligibility criteria, 134 studies remained for full text screening, and 24 studies were finally included in the review (Fig. 1). The general characteristics of included studies are summarized in Table 2. The review identified 20 single-country studies from 11 different countries, including Ethiopia (n = 5), India (n = 4), China (n = 2), Pakistan (n = 2), Argentina (n = 1), Brazil (n = 1), Burkina Faso (n = 1), Lao People’s Democratic Republic (n = 1), Malaysia (n = 1), Nigeria (n = 1) and South Africa (n = 1). In addition, the review included four studies that used multi-country data [25 countries (n = 1), 15 countries (n = 1), four countries (n = 1), and two countries (n = 1)]. The review did not identify any studies from the Middle East, North Africa or Central Asia. Among the 24 studies, 23 studies were model-based cost-effectiveness analyses, and one study was a cost-effectiveness analysis alongside an observational study. The majority of studies solely focused on maternal, newborn, or child health (n = 18, 75%). Other studies looked at infant and child nutrition (n = 2), health and nutrition (n = 1), nutrition and social protection (n = 2), and water and sanitation (n = 1). The most common outcomes were disability-adjusted life years (DALY) averted (n = 12), followed by deaths averted or lives saved (n = 7). Some studies measured outcomes as household expenditure or financial risk protection gained (n = 6) or other health outcomes, such as stunting averted or diarrhea averted (n = 6). No study measured any domain of child development including child language, cognitive, motor, social or emotional development. Despite this review only including studies from LMICs, the majority of studies were conducted by first authors based in high-income countries (HICs) (75%) mostly from the United States. More than half of included studies were conducted by a group of authors without anyone affiliated with institutions in the study setting (54%).

**Table 2:** Characteristics of included studies (n = 24)

Characteristics		Number	Percent (%)
Publication year	2010–2015	11	45.8
	2016–2020	11	45.8
	2021 up to 13 July	2	8.3
Country	Ethiopia	5	20.8
	India	4	16.7
	China	2	8.3
	Pakistan	2	8.3
	Argentina	1	4.2
	Brazil	1	4.2
	Burkina Faso	1	4.2
	Lao People’s Democratic Republic	1	4.2
	Malaysia	1	4.2
	Nigeria	1	4.2
	South Africa	1	4.2
	Multi-country	4	16.7
Study design	Modelling based on multiple data sources	23	95.8
	Observational study	1	4.2
Evaluation type	Cost-effectiveness analysis	24	100.0
Intervention	Health	18	75.0
	Nutrition	2	8.3
	Health and nutrition	1	4.2
	Nutrition and social protection	2	8.3
	Water, sanitation and hygiene	1	4.2
Perspective of analysis*	Healthcare provider/system/government	13	54.2
	Societal	8	33.3
	Household	3	12.5
	Not specifically stated	3	12.5
Outcome*	DALY averted	12	50.0
	Deaths averted/lives saved	7	29.2
	Household expenditure averted/financial risk protection gained	6	25.0
	Other health outcomes (i.e., stunting or diarrhea averted)	6	25.0
	Health or quality adjusted life years	2	8.3
	Life years gained	1	4.2
Country of first author's affiliation	United States	11	45.8
	Norway	3	12.5
	Switzerland	2	8.3
	United Kingdom	2	8.3
	Argentina	1	4.2
	Malaysia	1	4.2
	Brazil	1	4.2
	China	1	4.2
	United Kingdom, Burkina Faso	1	4.2
	United States, China	1	4.2
At least one author is based in the study setting	Yes	11	45.8
	No	13	54.2

*DALY* Disability-adjusted life year

^*^Multiple counts

### Equity Incorporation

**Fig. 2:**
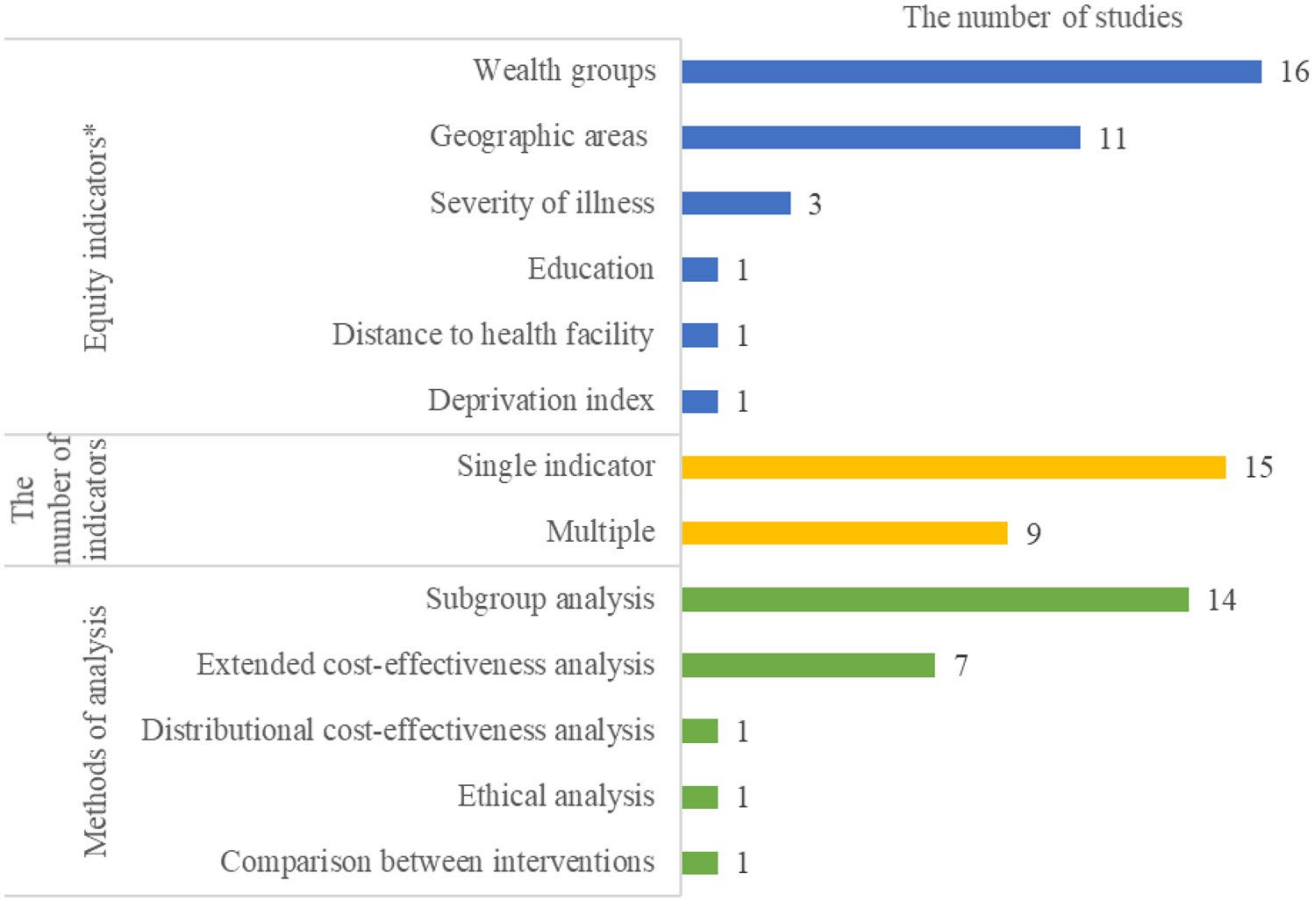
Equity characteristics of included studies (n = 24). *Multiple counts

The characteristics of equity measures and description of included studies are presented in Fig. 2 and Table 3. Most studies used wealth groups (n = 16, 67%) as equity indicators, followed by geographic areas (n = 11, 46%). The wealth groups, quintiles (n = 13) or deciles (n = 2), were mostly based on the Demographic and Health Surveys Wealth Index rank derived from household’s assets, materials used for housing construction and types of water access and sanitation facilities (Rutstein & Johnson, 2004). One study used the World Bank international poverty line of US$5.50 per day. Regarding equity measures, the majority of studies used one indicator (n = 15), while some studies used more than one indicator mostly using geographic areas and wealth groups together (n = 9). Subgroup analysis was the most common method used to incorporate equity into economic evaluations (n = 14, 59%), and seven studies used extended cost-effectiveness analyses.

**Table 3:** Description of included studies (n = 24)

Author, year	Country	Intervention vs. comparator	Design/Type/Perspective	Equity measures	Definition of equity measures	Methods of analysis	Findings
Health: Immunization							
(Rheingans et al., 2012)	25 countries	Rotavirus vaccination vs. No vaccination	Modelling CEA NS	Wealth groups, geographical regions	Wealth quintiles using the same method as DHS, states	Subgroup analyses	From US$28 in DR Congo to US$140 in Bangladesh per DALY averted (national) In all countries, the ICER was least favorable in the richest quintiles and the greatest potential benefit of vaccination was in the poorest quintiles Cost-effectiveness and benefits differed substantially among states, from over US$250/DALY averted in Kerala to less than US$60/DALY averted in Madhya Pradesh
(Verguet et al., 2013)	India, Ethiopia	Rotavirus vaccination vs. No vaccination	Modelling CEA Societal	Wealth groups	Income quintile	Extended CEA	Lives saved: 32,000 in India; 3700 in Ethiopia Total household expenditures averted per million infants: US$1.8 million in India; US$0.8 million in Ethiopia Total financial risk protection (for 1,000,000 households): US$16,000 in India and US$8000 in Ethiopia More lives would be saved among the bottom income quintile compared to the top income quintile
(Rheingans et al., 2014)	India	Rotavirus vaccination vs. No vaccination	Modelling CEA NS (assumed provider)	Geographic areas, wealth groups, severity of illness	Six geographic regions, wealth quintiles based on asset index, three high mortality states	Subgroup analyses	US$139 per DALY averted (national); Ranged from US$95 to US$298 depending on regions Rotavirus vaccines are most cost-effective for the poor living in high mortality regions and states. The lowest (most favorable) ratio in the high mortality regions
(Urueña et al., 2015)	Argentina	Rotavirus vaccination vs. No vaccination	Modelling CEA Health care system and societal	Geographic areas	Country-wide, Northeast and Northwest, where hospitalizations and deaths are more frequent	Subgroup analyses	US$3870 and US$1802 for monovalent rotavirus vaccine, and US$2414 and US$358 for pentavalent rotavirus vaccine per DALY averted, from the health care system and societal perspective, respectively. Northeast were US$1470 and US$636 for monovalent rotavirus vaccine, and US$913 and US$80 for pentavalent rotavirus vaccine ICERs were lower in the Northeast and Northwest for both vaccines. Health and economic benefits would be higher in the Northeast and Northwest regions
(Pecenka et al., 2015)	Ethiopia	Universal public finance in diarrheal treatment along with rotavirus vaccination vs. Treatment alone	Modelling CEA Provider and household (assumed)	Wealth groups	Wealth quintile (based on Central Statistical Agency [Ethiopia], DHS)	Extended CEA	Per US$1 million invested, diarrheal treatment saves 44 lives and averts US$115,000 in private expenditures. For the same investment, diarrheal treatment and rotavirus vaccination save 61 lives and avert US$150,000 in private expenditures Deaths averted: the interventions provide greater benefits to the poor Private expenditures averted: the wealthy tend to experience the greatest gains
(Loganathan et al., 2016)	Malaysia	Rotavirus vaccination vs. No vaccination	Modelling CEA Household	Wealth groups	Income quintiles (based on National Health and Morbidity Survey 2011)	Extended CEA	US$6 million savings to households annually. Rotavirus vaccination results in substantial reduction in rotavirus episodes and expenditure and provides financial risk protection to all income groups Poverty reduction benefits are concentrated amongst the poorest two income quintiles
(Dawkins et al., 2018)	Ethiopia	Pro-poor rotavirus vaccination vs. Standard vaccination	Modelling CEA Provider (assumed)	Wealth groups	Wealth quintiles (based on Central Statistical Agency Ethiopia, DHS)	Distributional CEA	US$69 per health-adjusted life year The pro-poor vaccine falls in the south-east ‘lose-win’ quadrant, demonstrating that relative to the standard vaccination program, the pro-poor vaccine has a positive impact on health equity despite its negative impact on total health
(Rheingans et al., 2018a)	Lao People’s Democratic Republic	Rotavirus vaccination vs. No vaccination	Modelling CEA NS	Geographic areas by wealth groups	Three regions, wealth quintiles based on a national asset index (DHS wealth index)	Subgroup analyses	US$140 per DALY averted (national). Ranged from US$124 to US$158 The ICER varies within region and is lowest (most cost-effective) in the poorer and poorest quintiles in all regions due to the higher burden of disease
(Rheingans et al., 2018b)	Pakistan	Rotavirus vaccination vs. No vaccination	Modelling CEA Donor and government	Geographic areas by wealth groups	Five regions, wealth quintiles based on an asset index (DHS wealth index)	Subgroup analyses	US$279 (GAVI), US$224 (government) per DALY averted at national level. Ranged from US$122 to US$594 The ICER varied within region and was lowest (most cost-effective) in the poorest quintiles in all regions Vaccination is most cost effective in high burden areas, and still highly cost-effective across all subpopulations
(Anderson et al., 2020)	Nigeria	Rotavirus vaccination vs. No vaccination	Modelling CEA Donor and government	Geographic areas by wealth groups	Six regional administrative units, wealth quintiles (defined by DHS wealth index)	Subgroup analyses	ICER per DALY averted: US$62 (GAVI), US$47 (government) at national level. Ranged from US$25 to US$64
(Driessen et al., 2015)	Ethiopia	(i) Measles routine immunization, (ii) immunization with financial incentives, (iii) Mass campaigns vs. No vaccination	Modelling CEA Societal	Wealth groups	Income quintile	Extended CEA	ICERs per death averted: US$303 (poorest)-1029 (richest) routine immunization, US$2318 (poorest)-1029 (richest) routine immunization supplemented by financial incentives, US$415 (poorest) -1291 (richest) supplemental immunization activities Household expenditures averted (thousands of $): US$59 (poorest)-9 (richest) routine immunization, US$167 (poorest)-9 (richest) routine immunization supplemented by financial incentives, US$481 (poorest)—73(richest) supplemental immunization activities
(Anderson et al., 2019)	Democratic Republic of Congo, Kenya, Zambia, Zimbabwe	Enterotoxigenic Escherichia coli and *Shigella* vaccination vs. No vaccination	Modelling CEA NS (assumed provider)	Geographic areas by wealth groups	Wealth quintiles (DHS wealth index), provincial areas	Subgroup analyses	Both vaccines were most cost-effective (lower ICERs) in lower and lowest quintiles of higher burden subnational areas in all countries, with few exceptions where middle and higher wealth subpopulation estimates were similar to the lowest two quintiles Enterotoxigenic Escherichia coli and Shigella vaccines are most cost effective when the most vulnerable and impoverished populations are vaccinated
(Luz et al., 2021)	Brazil	Infant whole-cell pertussis vaccination vs. Maternal acellular pertussis immunization plus infant vaccination	Modelling CEA Health system	Geographical areas	Three socio-demographically distinct Brazilian states	Subgroup analyses	US$3068 in Sao Paulo, US$2962 in Parana, and US$2022 in Bahia per DALY averted For all three Brazilian states, maternal acellular pertussis immunization led to higher costs but also saved infant lives and averted DALYs
Health: Pneumonia							
(Johansson et al., 2015)	Ethiopia	Publicly financed pneumococcal vaccination and pneumonia treatment vs. No intervention	Modelling CEA Health system, household	Wealth groups	Wealth quintile (based on DHS)	Extended CEA	Both programs save more lives among the poorest groups Wealthier people avert more private expenditures Household expenditures averted (US$ in 1000): 122 (poorest) to 71 (richest) from vaccines; 207 (poorest) to 480 (richest) from treatment Financial risk protection (US$ in 1000): 17 (poorest) to 1 (richest) from vaccines; 102 (poorest) to 7 (richest) from treatment Deaths averted: 1004 (poorest) to 152 (richest) from vaccines; 886 (poorest) to 134 (richest) from treatment
(Olsen et al., 2021)	Ethiopia	Scaling up coverage of community-based treatment of childhood pneumonia vs. Baseline	Modelling CEA Provider	Geographical areas	Eleven major regions	Subgroup analyses	The ICER per life year gained ranged from US$26 to US$196 Prioritizing regions with high under-five mortality rate is effective in reducing geographical inequalities
Health: Maternal and newborn							
(Hounton & Newlands, 2012)	Burkina Faso	Skilled care initiative vs. Status quo	Observational study CEA Societal	Education, distance to health facility	Education: no education, some level of education (at least primary school level) Distance: within 5 km of the closest health facility, more than 5 km	Subgroup analyses, Net-Benefit Framework	Int$170 for achieving one additional institutional delivery Varied significantly by covariates. By adjusting the intervention cost-effectiveness results to the covariates, we were able to identify distance to health facilities as an important determinant of the CEA
(Miljeteig et al., 2010)	India	Newborn treatment between gestational age weeks 28 and 32 vs. No intervention	Modelling CEA Not reported	Severity of illness	28 or 32 gestational age weeks	Kymlicka’s ethical case analysis	US$12–73 per QALY depending on gestational age weeks
Health: Hearing screening							
(Huang et al., 2012)	China	Universal vs. Targeted screening vs. No intervention	Modelling CEA Provider (assumed)	Geographic areas	Eight provinces	Comparison between interventions	Universal: More advantaged provinces Int$167,951–504,564 per DALYs averted; More advantaged provinces Int$18,057—106,497 per DALYs averted Targeted: More advantaged provinces Int$22,503–83,305 per DALYs averted; More advantaged provinces Int$3941–14,868 per DALYs averted Cost-effective in more advantaged provinces; not cost-effective in less advantaged provinces
Health and nutrition							
(Carrera et al., 2012)	14 countries and one province in Pakistan	Equity-focused vs. Mainstream approach	Modelling CEA Societal (assumed)	Deprivation (Lack of coverage)	Lack of coverage of interventions determined by geographical, economic, and sociocultural factors	Subgroup analyses	Lives saved: 97 in the most deprived populations; 61 in the least deprived populations per each $1 million invested Stunting averted: 279 cases averted in the most deprived populations; 188 cases averted in the least deprived populations per each $1 million invested 24% points of expenditure borne by families decreased for the equity-focused, 13% points decreased for the mainstream approach
Nutrition							
(Taylor et al., 2018)	South Africa	Donor human breast milk vs. Formula milk	Modelling CEA Health services	Severity of illness	4 birthweight groups (500-750 g, 751–1000 g, 1001–1250 g, and 1251–1500 g)	Subgroup analyses	US$619 per DALY averted was the worst‐case allocation scenario in terms of cost‐effectiveness (only giving donor milk to infants in the 500–750 g birthweight group for 14 days) Prioritizing infants in the lowest birthweight groups would save the most lives, whereas prioritizing infants in the highest birthweight groups would result in the highest cost savings
(Li et al., 2020)	China	Nutritional package vs. No intervention	Modelling CEA Provider (assumed)	Geographic areas by wealth groups	25 provinces in rural China. Poverty and non-poverty groups based on the international poverty line of $5.50/day	Extended CEA	¥785–23,324 per stunting case averted. The cost per stunting case averted would greatly vary across Chinese provinces and wealth groups
Nutrition and social protection							
(Plessow et al., 2016)	India	Price subsidies on fortified packaged infant cereals vs. No intervention	Modelling CEA Societal	Socio-economic status	Ten socio-economic strata determined by the deciles of a wealth index (based on DHS wealth index)	Subgroup analyses	The costs of the subsidies per DALY averted range from US$909 to US$3649. Return per DALY averted ranges between gains of US$1655 to a cost of US$411 Interventions targeted poorer households are most effective (target two poorest group: US$909 vs. target whole group: US$2473)
(Wieser et al., 2018)	Pakistan	Price subsidies on fortified packaged complementary foods vs. No intervention	Modelling CEA Societal	Socio-economic status	Ten socio-economic strata based on DHS, National Nutrition Survey	Subgroup analyses	Net saving of US$65–783 per DALY averted Most cost-effective intervention is a 20% subsidy for the poorest 20% of the population, with a net saving of $US783 million per DALY averted
Water and sanitation							
(Nandi et al., 2017)	India	Access to water and sanitation vs. Baseline	Modelling CEA Societal (assumed)	Wealth groups	Wealth quintile based on a composite index of asset ownership and living conditions	Extended CEA	Out-of-pocket expenditure averted was US$36,530 (richest) to US$586,765 (poorest); incremental cost to government was US$1,470,011 (richest) to US$3,198,001 (poorest); value of insurance was US$64 (richest) to US$7125 (poorest) when the coverage rates are separately increased across all Indian households randomly to a 95% level Out-of-pocket expenditure averted was US$33,799 (richest) to US$596,952 (poorest); incremental cost to government was US$1,569,839 (richest) to US$3,298,963 (poorest); value of insurance was US$66 (richest) to US$7185 (poorest) when the coverage rates are increased to at least 95% level separately within each state

*CEA* Cost-effectiveness analysis; *DALY* Disability-adjusted life year; *DHS* Demographic and Health Survey; *ICER* Incremental cost-effectiveness ratio; *NS* Not specified; *QALY* Quality-adjusted life year

### Summary of Findings

A summary of included studies is presented in Table 3. Among eight out of 10 studies, rotavirus vaccinations were found to be more beneficial to the disadvantaged groups than less disadvantaged groups in terms of geographic areas, wealth, or severity of illness (Anderson et al., 2020; Dawkins et al., 2018; Loganathan et al., 2016; Pecenka et al., 2015; Rheingans et al., 2014; Rheingans et al., 2018a; Rheingans et al., 2018b; Rheingans et al., 2012; Urueña et al., 2015; Verguet et al., 2013). An extended cost-effectiveness analysis from Ethiopia reported mixed findings in terms of equity and cost-effectiveness as they varied across different measles vaccination strategies (Driessen et al., 2015). Enterotoxigenic Escherichia coli and *Shigella* vaccination were found to be most cost-effective when the most vulnerable and impoverished populations were vaccinated in four countries in sub-Saharan Africa (Anderson et al., 2019). Luz et al., found that maternal acellular pertussis immunization led to higher costs, but also saved infant lives and averted DALYs in Brazilian states (Luz et al., 2021). An extended cost-effectiveness analysis from Ethiopia found that both pneumococcal vaccine and pneumonia treatment would save more lives among the poorest groups, but averted more private expenditure among wealthier people (Johansson et al., 2015). Another study looking at pneumonia treatment concluded that prioritizing regions with high mortality rates for children under the age of 5 is effective in reducing geographical inequalities in Ethiopia (Olsen et al., 2021). The equity-related conclusions were not clear in two studies addressing skilled care initiative from Burkina Faso (Hounton & Newlands, 2012) and newborn treatment in India (Miljeteig et al., 2010) as findings varied by study outcomes and other covariates. A study from China concluded that hearing screenings for neonates were cost-effective only in more advantaged provinces but not in less advantaged provinces (Huang et al., 2012). A multi-country study reported that an equity-focused approach to child survival, health, and nutrition could save more lives, avert stunting, and reduce expenditure by families in the most deprived populations, compared to the least deprived populations (Carrera et al., 2012). A study of donor human breastmilk from South Africa reported that prioritizing infants in the lowest birthweight groups would save the most lives, whereas prioritizing infants in the highest birthweight groups would result in the highest cost savings (Taylor et al., 2018). In China, the cost per stunting case averted through a nutritional package varied across provinces and wealth groups, but the authors concluded that the cost would be lower for children living under the poverty line in most provinces (Li et al., 2020). The price subsidies on fortified packaged infant cereals which targeted poorer households were cost-effective in India, or even cost-saving for the poorest households in Pakistan (Plessow et al., 2016; Wieser et al., 2018). A study from India examining scaling up access to piped water and improved sanitation found that the poorest group gained greater child health and financial benefits (Nandi et al., 2017).

### Equity Impacts

Most studies reported that early childhood development interventions improved equity, with more benefits observed among more disadvantaged groups compared to less disadvantaged groups (n = 15, 63%) (Table 4) (Anderson et al., 2019; Carrera et al., 2012; Dawkins et al., 2018; Loganathan et al., 2016; Nandi et al., 2017; Olsen et al., 2021; Plessow et al., 2016; Rheingans et al., 2014; Rheingans et al., 2018a; Rheingans et al., 2018b; Rheingans et al., 2012; Taylor et al., 2018; Urueña et al., 2015; Verguet et al., 2013; Wieser et al., 2018). Among them, interventions from nine studies were found to be more cost-effective or cost-saving in the disadvantaged groups compared to less disadvantaged groups (Anderson et al., 2019; Carrera et al., 2012; Plessow et al., 2016; Rheingans et al., 2014; Rheingans et al., 2018a; Rheingans et al., 2018b; Rheingans et al., 2012; Urueña et al., 2015; Wieser et al., 2018). In contrast, two studies reported that interventions were less cost-effective in the disadvantaged group though the interventions improved equity (Dawkins et al., 2018; Taylor et al., 2018). Around 30% of studies reported mixed findings as the results varied by other variables and study outcomes (Anderson et al., 2020; Driessen et al., 2015; Hounton & Newlands, 2012; Johansson et al., 2015; Li et al., 2020; Miljeteig et al., 2010; Pecenka et al., 2015). In total, one study reported that the intervention was only cost-effective in more advantaged provinces but not in less advantaged provinces (Huang et al., 2012).

**Table 4:** Equity impact

Equity impact		Number	Percent (%)
Pro-disadvantaged	More cost-effective or cost-saving in the disadvantaged groups	9	37.5
	No conclusion of cost-effectiveness	4	16.7
	Less cost-effective in the disadvantaged groups	2	8.3
	Subtotal	15	62.5
Mixed as it varied by variables or outcomes		7	29.2
Not pro-disadvantaged, not cost-effective		1	4.2
No conclusion		1	4.2

## Discussion

This scoping review examined how equity is integrated into the economic evaluations of early childhood development interventions in LMICs, and synthesized the study characteristics and findings. The identified 24 articles covered health, nutrition, social protection, and water, sanitation and hygiene interventions from 37 LMICs, and examined their cost-effectiveness and equity. The equity issues were mostly measured by household wealth and geographic areas, and equity findings were presented by subgroup analyses. Overall, early childhood development was mostly addressed through childhood immunization alone rather than multi-sectoral interventions from LMICs in the regions of Asia and Africa. Most studies were conducted by research teams from HICs. More than half of studies reported that the interventions improved equity as disadvantaged groups gained more benefits than less disadvantaged groups.

Wealth groups were the most common equity indicators followed by geographic areas in included studies in this review. Previous review papers also reported similar findings that socioeconomic status was the most common equity criterion in health economic evaluations (Avanceña & Prosser, 2021; Yang et al., 2021). Yang and colleagues found that socioeconomic status was categorized mostly based on wealth quintiles, and place of residence were the next common equity criterion (Yang et al., 2021). In another review, race/ethnicity and geography were also identified as common equity criteria (Avanceña & Prosser, 2021). Wealth is one of the most common social determinants of focus by policy makers, thus that could be the reason why several studies chose to use wealth groups to look at equity issues. One study adopted a deprivation index considering geographical, economic, and sociocultural factors (Carrera et al., 2012). Measuring equity based on multiple factors may provide a broader picture of distribution of health benefits and their cost-effectiveness; however, the feasibility of data collection should also be considered. Factors that imply inequity could be also context-specific, considering differences in settings and challenges.

A number of methods were applied to present equity findings. Subgroup analysis was the most common method followed by extended cost-effectiveness analysis. Presenting cost-effectiveness results by subgroups has previously been found to be the most common method, described as the straightforward way to present the different impacts of health interventions across populations in one review (Yang et al., 2021). The extended cost-effectiveness analysis or distributional cost-effectiveness analysis approach was used less commonly, and the previous review also indicated that the knowledge and application of these methods were not yet widespread in LMICs (Yang et al., 2021). Even in HICs, most research focus on effectiveness of health policies and programs without much consideration of equity. The advanced methods can provide additional information on financial risk protection benefits and tradeoffs between improving total health and reducing inequality from interventions. Addressing equity requires careful research planning and implementation, which need to be context-specific based on health systems.

Effective early childhood development interventions require collective work across sectors to ensure that every child reaches their full potential in physical, cognitive, and psychosocial development, yet this review only identified a few studies with multi-sectoral interventions. Additionally, no study in our review measured any domain of child development including language, cognitive, motor, social or emotional development as outcomes. Overall, we discovered that current research trends heavily focused on childhood immunization interventions in Asia and Africa regions. A large proportion of immunization studies reflect global efforts to reduce preventable deaths and increase child survival over the past few decades. Beyond survival, the global agenda is now also focused on enabling children to thrive. The WHO’s Nurturing Care Framework highlights that children need nurturing care which is the conditions that promote health, nutrition, security, safety, responsive caregiving and early learning to develop to their full potential (World Health Organization et al., 2018). A multi-sectoral framework to promote child development has been also proposed, highlighting the need for interventions through services and programs of several sectors in the context of a supportive environment of policies, coordination, and financing (Richter et al., 2017). Furthermore, combining key interventions as packages of care for child development has been suggested including complementary feeding education and provision, micronutrient supplementation, and integrated responsive caregiving and early learning interventions (Vaivada et al., 2022).

Research in early childhood development has advanced since 2000, and over 4000 publications were identified between 2000 and 2014 (Black et al., 2017). However, our review identified only 24 publications considering equity in economic evaluations of early childhood development in LMICs since 2000, which highlights the need for more investment in this field. Overall, 63% of included studies reported that early childhood development interventions improved equity, with more benefits to disadvantaged groups. Focusing solely on cost-effectiveness of interventions may not provide a full picture of interventions’ impacts, thus considering equity would be more desirable for informed decision-making. Equity consideration requires more emphasis on the most disadvantaged children to ensure their full development, and to achieve social justice and realize the United Nations Sustainable Development Goals globally (United Nations, 2015).

Even though included studies focused on early childhood development in LMICs, most studies were conducted by researchers based in HICs. Researchers from LMICs have greater knowledge and lived experience about contexts and cultural factors in specific LMICs, and can provide deeper insights into potential solutions (Nafade et al., 2019), thus their involvement in research is paramount. However, the underrepresentation of LMICs in global health has been identified in terms of authorships, conference participations, and editorial boards in previous studies (Iyer, 2018; Nafade et al., 2019; Velin et al., 2021). The data showed that 35% of the authors of research articles were affiliated with LMICs (Iyer, 2018), 11% of journal editors were women based in LMICs (Nafade et al., 2019), 4% of global health conferences were hosted in LMICs and 39% of attendees were from LMICs (Velin et al., 2021). Research resources, infrastructure, and funding are dominated by HICs, which leads to less involvement from LMICs in shaping the global health agenda, priority setting, and policies. Considering that challenges in LMICs take a huge part in global health, more efforts to promote equity, diversity, and inclusion are required to achieve health for all.

The few relevant studies conclude that there is a need for more economic evidence to promote child development with equity considerations. First, technical guidance to support design, implementation, and evaluation of equity-informed economic evaluations in LMICs would be helpful. Second, the few identified multi-sectoral interventions indicate that strengthening a multi-sectoral approach is required to ensure holistic child development. Collective work across multiple sectors including health, nutrition, security and safety, responsive caregiving, and education can maximize the impact of interventions to meet diverse needs of children. Third, providing more technical and financial support to researchers in LMICs will support context-based evidence generation. Lastly, policy makers will also need clear and informed guidance on translating evidence to refine child development strategies and programs.

This review has some limitations. As a scoping review, we did not conduct quality assessment and quantitative synthesis of results. Given breadth of early childhood development interventions covered, rather than a quantitative synthesis, this review aimed to provide an overview of existing evidence on how equity is integrated into economic evaluations in research in LMICs, and equity findings. Additionally, the search was restricted to English literature in scientific journals, which may have missed some studies. Lastly, we acknowledge the limitation of having one author conducting study selection and data extraction, but note that other authors were involved in discussions and decisions to address any uncertainties.

## Conclusions

Every child has the right to reach their full potential, and equity is key to ensure that. Considering equity in economic evaluation could provide a broader picture to make more informed-decisions in priority setting. The small number of relevant studies in the review highlights that more emphasis on equity integration into economic evaluation, coordinated work across multiple sectors, and strong involvement of researchers based in LMICs, are necessary to improve child development.

## Supplementary Information

Below is the link to the electronic supplementary material.

Supplementary file1 (DOCX 24 KB)

## Data Availability

All data generated or analyzed during this study are included in this article.

## Abbreviations

DALY: Disability-adjusted life year
GNI: Gross national income
HICs: High-income countries
LMICs: Low-and middle-income countries
PRISMA-ScR: Preferred reporting items for systematic reviews and meta-analyses extension for scoping reviews
WHO: World health organization
