# Supplementary material for: Equity in Economic Evaluations of Early Childhood Development Interventions in Low-and Middle-Income Countries: Scoping Review
Source: Matern Child Health J. 2023 Apr 10;27(6):1009–29. doi: 10.1007/s10995-023-03650-3 (PMC10160157; doi:10.1007/s10995-023-03650-3)
Supplement: Supplementary file 1 — Supplementary file1 (DOCX 24 KB) [file 10995_2023_3650_MOESM1_ESM.docx]

**Electronic Supplementary Material**

**Equity in economic evaluations of early childhood development interventions in low-and middle-income countries: scoping review**

# Supplementary Table 1. Search strategy for Ovid MEDLINE

| **#** | **Searches** |
| --- | --- |
| 1 | exp Health Equity/ or exp Social Justice/ or exp Vulnerable Populations/ |
| 2 | exp Health Status Disparities/ or exp Socioeconomic Factors/ or exp Healthcare Disparities/ |
| 3 | (equit* or inequalit* or disparit* or fair).mp. |
| 4 | or/1-3 |
| 5 | exp Cost-Benefit Analysis/ |
| 6 | (cost* benefit* or cost* effective* or economic evaluation or cost* utilit*).mp. |
| 7 | or/5-6 |
| 8 | ((cognitive or language or motor or social-emotional or socio-emotional) adj (development or stimulation)).mp. |
| 9 | exp Health Education/ or exp Community Health Services/ or exp Health Services Accessibility/ or exp Mental Health Services/ or exp Health Services Research/ or exp Health Behavior/ or exp Mental Health/ or exp Preventive Health Services/ or exp Health/ or exp Health Risk Behaviors/ or exp Primary Health Care/ or exp Comprehensive Health Care/ or exp Community Mental Health Services/ or exp "Health Services Needs and Demand"/ or exp Health Promotion/ or exp Health Communication/ or exp Health Knowledge, Attitudes, Practice/ or exp Global Health/ or exp Health Services/ or exp Public Health/ |
| 10 | exp Sanitation/ or exp Hygiene/ |
| 11 | exp Vaccines/ or exp Vaccination/ or exp Malaria Vaccines/ |
| 12 | (health or immunization or immunisation or vaccine or vaccination* or WASH or sanitation or hygiene or mental health or mental disorder* or depression).mp. |
| 13 | exp Protein-Energy Malnutrition/ or exp Malnutrition/ or exp Severe Acute Malnutrition/ or exp Nutrition Policy/ |
| 14 | (nutrition or malnutrition or stunting or undernutrition or overweight or obesity or obese or breastfeeding or micronutrient*).mp. |
| 15 | exp Education/ |
| 16 | (home visit* or home stimulation or violence* or maltreatment or abuse).mp. |
| 17 | (cash transfer* or voucher* or microcredit* or subsidy or subsidies or social protection or insurance or safety net).mp. |
| 18 | or/8-17 |
| 19 | exp Infant/ or exp Infant Care/ or exp Infant, Newborn/ or exp Child Care/ or exp Child/ or exp Child, Preschool/ or exp Pregnant Women/ or exp Parents/ or exp Caregivers/ |
| 20 | (newborn* or infant* or child* or maternal or paternal or pregnan* or parent* or caregiver* or mother* or father*).mp. |
| 21 | or/19-20 |
| 22 | 18 and 21 |
| 23 | exp Child Development/ or exp Early Intervention, Educational/ |
| 24 | (early childhood development or ECD or child development or childhood development or brain development).mp. |
| 25 | exp Child Health/ or exp Child Health Services/ or exp Infant Health/ or exp Maternal-Child Health Services/ or exp Maternal Health Services/ or exp Reproductive Health/ or exp Reproductive Health Services/ or exp Women's Health Services/ or exp Women's Health/ or exp Sexual Health/ or exp Family Health/ |
| 26 | exp Child Nutrition Disorders/ or exp Child Nutrition Sciences/ or exp Infant Nutrition Disorders/ |
| 27 | (school* or preschool* or early learning or education).mp. |
| 28 | exp Parenting/ or exp Child Protective Services/ or exp Child Abuse, Sexual/ or exp Child Welfare/ or exp Child Abuse/ |
| 29 | (child protection or caregiving or parenting or parent support or responsive caregiving).mp. |
| 30 | or/23-29 |
| 31 | 22 or 30 |
| 32 | exp Developing Countries/ |
| 33 | (LMIC or ((developing or low-income or middle-income or lowincome or middleincome) adj (country or countries or nation or nations))).mp. |
| 34 | (Afghanistan or Afghan or Albania* or Algeria* or American Samoa* or Andorra* or Angola* or "Antigua and Barbuda" or Argentina* or Armenia* or Aruba* or Azerbaijan* or Bahrain* or Bangladesh* or Barbados or Belarus* or Belize* or Benin* or Bhutan* or Bolivia* or "Bosnia and Herzegovina" or Bosnian or Botswana* or Brazil* or British Virgin Island* or Brunei* or Bulgaria* or Burkina Faso or Burundi* or Cabo Verde or Cape Verde* or Cambodia* or Cameroon* or Cayman Island* or Central African Republic or Chad* or Chile* or China or Chinese or Colombia* or Comoros or Comoran or Congo or Congolese or Costa Rica* or Cote d Ivoire or Ivory Coast or Croatia* or Cuba* or Curacao or Cyprus or Cypriot or Czech or Djibouti* or Dominica or Dominican or Ecuador* or Egypt* or El Salvador or Salvadoran or Equatorial Guinea* or Eritrea* or Estonia* or Eswatini or Swazi* or Ethiopia* or Fiji* or French Polynesia* or Gabon* or Gambia* or Georgia* or Ghana* or Gibraltar* or Greece or Greek or Grenada or Guam or Guatemala* or Guinea* or Guinea-Bissau* or Guyana or Guyanese or Haiti* or Hondura# or Hungary or Hungarian or India* or Indonesia* or Iran* or Iraq* or Isle of Man or Jamaica* or Jordan* or Kazakhstan* or Kenya* or Kiribati or Korea* or Kosovo or Kosovar or Kyrgyz* or Kirghiz or Lao* or Latvia* or Lebanon or Lebanese or Lesotho or Mosotho or Basotho or Liberia* or Libya* or Liechtenstein* or Lithuania* or Maca# or Madagascar or Malagasy or Malawi* or Malaysia or Maldiv* or Mali* or Malta or Maltese or Marshall Island* or Mauritania* or Mauritius or Mauritian or Mexico or Mexican or Micronesia* or Moldova* or Monaco or Monegasque or Mongolia* or Montenegro or Morocco or Mozambique or Myanmar* or Burma or Burmese or Namibia* or Nauru* or Nepal* or New Caledonia* or Nicaragua* or Niger* or Nigeria* or North Macedonia* or Northern Mariana Island* or Oman* or Pakistan* or Palau* or Panama* or Papua New Guinea* or Paraguay* or Peru* or Philippines or Filipino or Poland or Polish or Portugal or Portuguese or Puerto Rico or Puerto Rican or Romania* or Russia* or Rwanda* or Samoa* or San Marino or San Marinese or "Sao Tome and Principe" or Sao Tomean or Saudi Arabia* or Senegal* or Serbia* or Seychelles or Seychellois or Sierra Leone* or Sint Maarten* or Slovak* or Slovenia* or Solomon Island* or Somalia* or South Africa* or South Sudan* or Sri Lanka* or "St Kitts and Nevis" or "Kittian and Nevisian" or St Lucia* or St Martin* or "St Vincent and the Grenadines" or Vincentian or Sudan* or Suriname* or Syria* or Tajikistan* or Tajik or Tadzhik or Tanzania* or Thailand or Thai or Timor Leste or Togo* or Tonga* or "Trinidad and Tobago" or Tunisia* or Turkey or Turkish or Turkmenistan* or "Turks and Caicos Island*" or Tuvalu* or Uganda* or Ukrain* or Uruguay* or Uzbekistan* or Vanuatu or Venezuela* or Vietnam* or "West Bank and Gaza" or Palestinian or Yemen* or Zambia* or Zimbabwe*).mp. |
| 35 | or/32-34 |
| 36 | 4 and 7 and 31 and 35 |
| 37 | limit 36 to yr="2000 -Current" |
| 38 | (extended cost-effectiveness analysis or distributional cost-effectiveness analysis).mp. |
| 39 | 31 and 35 and 38 |
| 40 | 37 or 39 |
